# *Veratrum parviflorum*: An Underexplored Source for Bioactive Steroidal Alkaloids

**DOI:** 10.3390/molecules27165349

**Published:** 2022-08-22

**Authors:** Jared T. Seale, Owen M. McDougal

**Affiliations:** Department of Chemistry and Biochemistry, Boise State University, Boise, ID 83725, USA

**Keywords:** *Veratrum*, steroidal alkaloids, Hedgehog signaling pathway, cyclopamine, cancer

## Abstract

Plants of the *Veratrum* genus have been used throughout history for their emetic properties, rheumatism, and for the treatment of high blood pressure. However, inadvertent consumption of these plants, which resemble wild ramps, induces life-threatening side effects attributable to an abundance of steroidal alkaloids. Several of the steroidal alkaloids from *Veratrum* spp. have been investigated for their ability to antagonize the Hedgehog (Hh) signaling pathway, a key pathway for embryonic development and cell proliferation. Uncontrolled activation of this pathway is linked to the development of various cancers; most notably, basal cell carcinoma and acute myeloid leukemia. Additional investigation of *Veratrum* spp. may lead to the identification of novel alkaloids with the potential to serve as chemotherapeutics. *V. parviflorum* is a relatively uncommon species of *Veratrum* that resides in the southeastern regions of North America. The phytochemical profile of this plant remains largely unexplored; however, bioactive steroidal alkaloids, including cyclopamine, veratramine, veratridine, and verazine were identified in its extract. The structural elucidation and bioactivity assessment of steroidal alkaloids in lesser abundance within the extract of *V. parviflorum* may yield potent Hh pathway inhibitors. This review seeks to consolidate the botanical and phytochemical information regarding *V. parviflorum.*

## 1. Introduction

Most modern therapeutics have originated from a treasure trove of secondary metabolites extracted from natural products of terrestrial or marine origin. An estimated 70,000 plant species have been used throughout history for medicinal purposes and more than 3000 plants are reported to contain compounds with anticancer properties [1,2]. The *Veratrum californicum* derived the steroidal alkaloid, cyclopamine; it was first isolated in 1965 and later identified as an inhibitor of the protein Smoothened (Smo), which is a critical protein in the Hedgehog signaling pathway [3,4]. Since this discovery, a new class of Food and Drug Administration (FDA) approved chemotherapeutics called Hedgehog pathway inhibitors have been developed for the treatment of cancers; most prominently, for basal cell carcinoma and acute myeloid leukemia [5]. Plants from the genus *Veratrum*, including *V. viride*, *V. album*, *V. nigrum*, and *V. californicum* have been extensively studied; they are found to be rich sources for unique steroidal alkaloids (>100 alkaloids/plant), with approximately 20% of these secondary metabolites being characterized [6]. Here, we present a consolidated review of the morphological, ecological, and phytochemical information regarding the sparsely studied *Veratrum* spp., *V. parviflorum*.

### 1.1. Background

#### 1.1.1. *Veratrum* Genus

**Figure 1:**
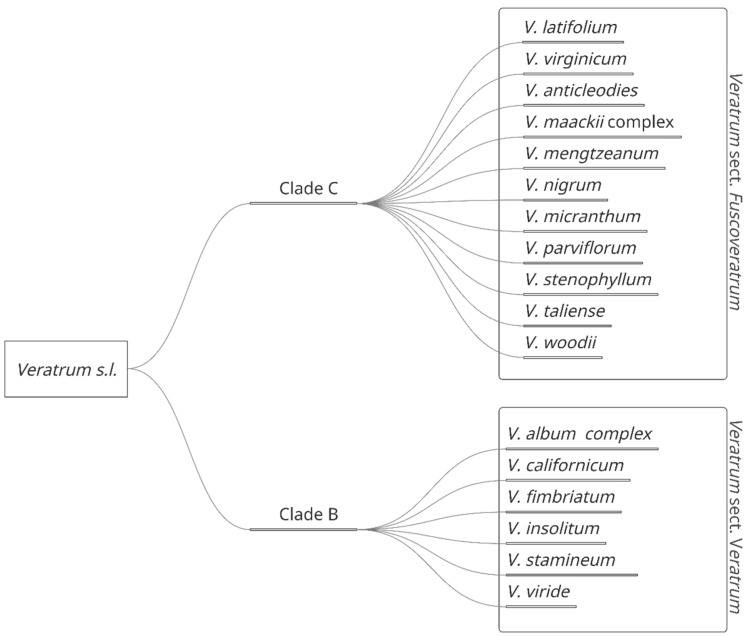
*Veratrum* spp. separated by classification in *Veratrum* sect. *Fuscoveratrum* and *Veratrum* sect. *Veratrum*.

The *Veratrum* genus is comprised of perennial flowering herbs located predominantly in the Northern hemisphere [7]. These plants are found throughout temperate regions of North America and northern temperate to arctic regions in Eurasia [7]. Depending on the taxonomic treatment, the number of species varies between 17–45 species. These can be divided into four species complexes: *V. album* L., *V. nigrum* L., *V. mackii* Regal, and *V. viride* Aiton [7,8]. Wide variability in taxonomic treatment may be attributed to a dissimilarity in morphology, including leaves, tepals, and perigonal nectaries, and habitats, including rocky tundra, bogs, meadows, riverbanks, swamps, and deciduous forest slopes [7]. *Veratrum* may be further divided into two major sections based on gynoecia characteristics: *Veratrum* sect. *Veratrum* [Clade B] and *Veratrum* sect. *Fuscoveratrum* [Clade C] (Figure 1).

#### 1.1.2. Medicinal Relevance

Traditional medicines have utilized *Veratum* spp. plants as a source of therapeutically active compounds for centuries [6,9]. Chinese medicine utilized *V. nigrum* in a medicinal concoction, referred to as Li-lu, to treat conditions including aphasia resulting from apoplexy, wind-type dysentery, jaundice, scabies, and chronic malaria [10]. The roots and rhizome of *V. album* subsp. *lobelianum* are described in the first Pharmacopoeia Rossica as a traditional Russian medicine that is made into a tincture or ointment for the treatment of head lice, scabies, neuralgic and rheumatic pain, eczema, or fevers [11]. This plant has also been used as an antiparasitic in cattle against hypodermatosis [11]. *V. album* has seen widespread use in Eurasia [12]. The Greeks used a powdered form of *V. album* to induce sneezing and for psychological diseases such as depression and epilepsy [12,13]. An alcohol extract of *V.* *album’s* roots was used in Italy as an antirheumatic [12]. In Iranian folk tradition, *V. album* root, pulverized into a paste, was used to relive headache and neuralgic pain [12]. In North America, Native American tribes, including the Shoshone, Bella Coola, Cherokee, Gitksan, Haisla, Hanaksiala, Iroquois, Kitasoo, Okanagan-Colville, Oweekeno, Quinault, Salish Thompson, and Tsimishian used the crushed roots of *V. viride* as an antirheumatic to treat snake bite wounds, to make a tea for venereal diseases, and as an analgesic for sore throats and colds [6,12]. Table 1 presents a comparison of several *Veratrum* spp., including the identified steroidal alkaloids and traditional medical applications.

## 2. *Veratrum parviflorum*

### 2.1. Taxonomy and Physical Characteristics

*Veratrum (Melanthium) parviflorum*, commonly known as mountain bunchflower, has a complex history regarding its classification in the *Veratrum* and *Melanthium* genera due to variation in the morphological constraints set by botanists [7,16]. To provide a more defined taxonomy of *Veratrum* spp., the nuclear ribosomal internal transcribed spacers (ITS) were analyzed and correlated to traditional taxonomic classifications, including flower color and geographical location [7]. The strict and bootstrap consensus trees were almost identical, except for *V. parviflorum*. The strict consensus suggested that *V. parviflorum* formed a subclade and was sister to *V. latifolium*, *V. virginicum*, and *V. woodii*; whereas the bootstrap consensus suggested that this species falls outside of the clade, forming a polytomy with the *V. maackii* and *V. micranthum* complexes [7].

**Figure 2:**
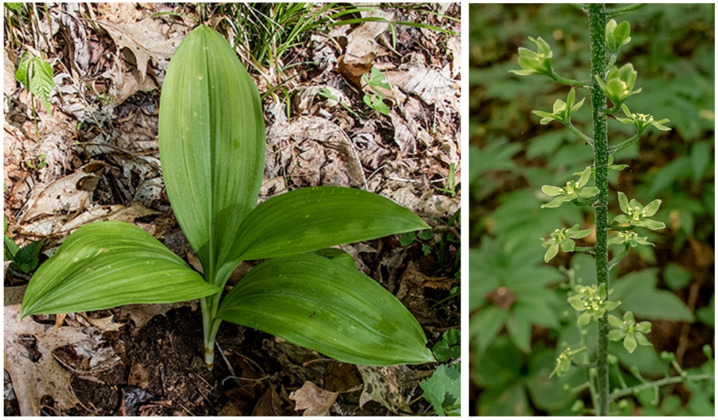
*V. parviflorum* in situ before blooming (**left**). In the later stages of growth, a stem protrudes from the base of the plant and blooms with pale green flowers (**right**) [19].

*V. parviflorum* is identified in nature using defined morphological traits. The stem is slender and 2 to 5 feet tall [15,17]. A pseudostem is formed by the overlapping sheaths of the leaves, which are broad (2–4 inches wide), petiolate, obscurely plicate, and have a blue tint adaxially [7,16,18]. The tepals are pale green to olive green, narrowly rhombic oblanceolate, with entire margins, gradually attenuated at base, filaments adnate, gland bilobed, diffuse, and dark (Figure 2) [7].

### 2.2. Geographic Location and Herbivory

*V. parviflorum* is found in the southeastern regions of North America [15,16,17,18,20]. This species grows in rich deciduous forests (800–2030 m) in the mid-Appalachians, including parts of Alabama, Georgia, Kentucky, North Carolina, South Carolina, Tennessee, Virginia, and West Virginia [15,16]. The states of Alabama, Kentucky, and West Virginia have classified the conservation status of *V. parviflorum* as critically imperiled (S1), imperiled (S2), and vulnerable (S3), respectively [18,20]. This plant is most easily discovered in the spring when it reaches peak germination [21]. Unfortunately, *V. parviflorum* growth season coincides with wild ramps (*Allium tricoccum*); furthermore, it has led to the accidental ingestion of *V. parviflorum*, resulting in cardiac and gastrointestinal toxicity [15]. Despite inducing toxic effects when ingested by humans, white-tailed deer have been observed to consume the entire inflorescences of this species [16,22].

### 2.3. Toxicity

Cases of *Veratrum* poisoning are found extensively within the literature; however, *V. parviflorum* has only been implicated in one case of poisoning in the United States [15]. Symptoms of *Veratrum* poisoning generally include nausea, vomiting, diarrhea, hypotension, bradycardia, hypopnea, paresthesia, or death if medical attention is not received [15,23,24,25,26,27,28,29,30,31,32]. Treatment for *Veratrum* poisoning is generally symptomatic and supportive; it may include the administration of atropine, intravenous fluids, vasopressors, activated charcoal, and promethazine [15,23,24,25,26,27,28,29,30,32]. Although digoxin immune Fab has been used for treating symptoms of cardiotoxicity similar to those observed in cases of *Veratrum* poisoning, it has been suggested that medical providers should not unnecessarily administer DigiFab^TM^ as they do not bind steroidal alkaloids extracted from *V. viride* [32]. Furthermore, Multigent^TM^ digoxin immunoassay reagent antibodies demonstrated cross-reactivity with the alkaloids; this resulted in false-positive tests [32]. Table 2 presents a summary of treatments for several cases of *Veratrum* poisoning.

The cardiotoxic effects from consuming *Veratrum* spp. are primarily due to the steroidal alkaloids produced by the plant [33]. These steroidal alkaloids are present throughout the plant; however, the roots and rhizomes contain higher concentrations than the leaves [31,34]. *Veratrum* steroidal alkaloids are recognized for their tendency to bind to the type 2 receptor site of voltage-gated sodium ion channels in vertebrate organisms [33,35]. Once bound, the resting membrane potential is depolarized, causing excitable membranes to fire repetitively [33,35,36]. Symptoms including bradycardia and hypotension are caused by the alkaloids interacting with cardiac receptors in the left ventricle posterior wall and the baroreceptor area of the coronary sinus; while depolarization in the vagus nerve can induce bradycardia, hypotension, and dyspnea [33,37]. Additional symptoms resulting from depolarization of the nerve cells may comprise of paresthesia, numbness, and vomiting [33]. The triad of symptoms, including bradycardia, hypotension, and dyspnea, caused by *Veratrum* poisoning is referred to as a Bezold–Jarisch reflex [6,33,38,39].

**Figure 3:**
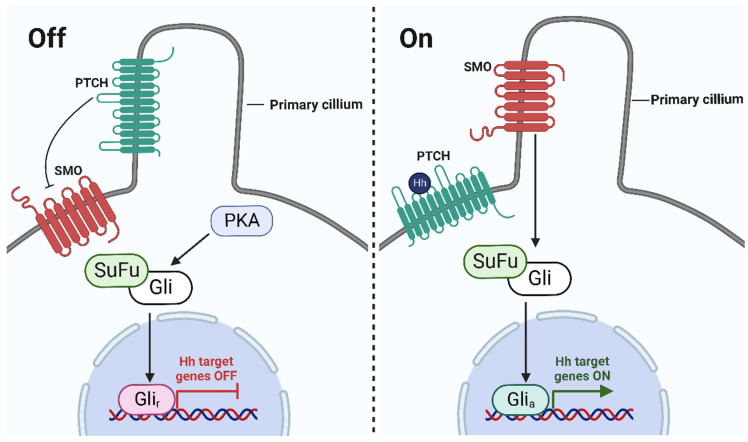
Schematic of the Hedgehog signaling pathway. In the absence of Hedgehog (Hh) ligands (**left**), Patched (PTCH) inhibits the G protein-coupled receptor, SMO. Protein kinase A (PKA) phosphorylates glioma-associated (Gli) transcription factors, which then undergo proteolytic cleavage from the suppressor of fused (SuFu) to generate the repressor form (Gli_r_). Gli_r_ hinders transcription of the Hh genes and turns the pathway off. In the presence of Hh ligands (**right**), PTCH is bound by the Hh ligand, resulting in the phosphorylation of SMO. Gli transcription factors dissociate from SuFu and generate the activator form (Gli_a_). Gli_a_ promotes the transcription of the Hh genes and turns the pathway on. In the presence of a Hh pathway inhibitor such as cyclopamine, SMO will remain inactivated and PKA will phosphorylate Gli transcription factors; thus, this will generate the Gli_r_ that inhibits transcription [46]. (Graphic created with BioRender.com.)

*Veratrum* steroidal alkaloids are recognized as antagonistic to the Hedgehog signaling pathway (Figure 3) [40,41,42]. In the late 1950s, sheep herders in the south-central and southwestern alpine meadows of Idaho observed that 1–25% of their lambs were born with cyclopean-type developmental defects [43]. These malformations were originally thought to be congenital; however, further investigations revealed that they resulted from pregnant ewes feeding on *V. californicum* between the 10th and 15th days of gestation [43,44]. The *Veratrum* steroidal alkaloids cyclopamine, jervine, cycloposine, and veratrosine were identified as the causative teratogenic agents [45]. *Veratrum* steroidal alkaloids exert teratogenic effects via antagonism of the Hedgehog signaling pathway by directly binding to the transmembrane protein Smoothened (Smo) [4,40]. The binding of the small molecule to Smo takes place in the extracellular pocket of Smo, inhibiting activation by membrane sterols [46].

### 2.4. Phytochemistry

There is a lack of published information regarding the steroidal alkaloid content in *V. parviflorum*. Currently, only four alkaloids have been identified in an extract of *V. parviflorum* root and rhizome [15]. The four alkaloids, including cyclopamine, veratramine, verazine, and veratridine, are not novel to the *Veratrum* genus and have been investigated for a variety of bioactive properties. Challenges to the structural elucidation of *Veratrum* steroidal alkaloids are largely due to the complexity of the molecules and presence of isomers. Many alkaloids cannot be differentiated by chemical formula and require full characterization, unless a commercially available standard can be purchased. A variety of instrumentation may be used to identify the structures of unknown steroidal alkaloids, including nuclear magnetic resonance (NMR) spectroscopy, infrared spectroscopy, ultraviolet spectroscopy, and liquid chromatography mass spectrometry [4,14,15,34,41].

#### 2.4.1. Cyclopamine

**Figure 4:**
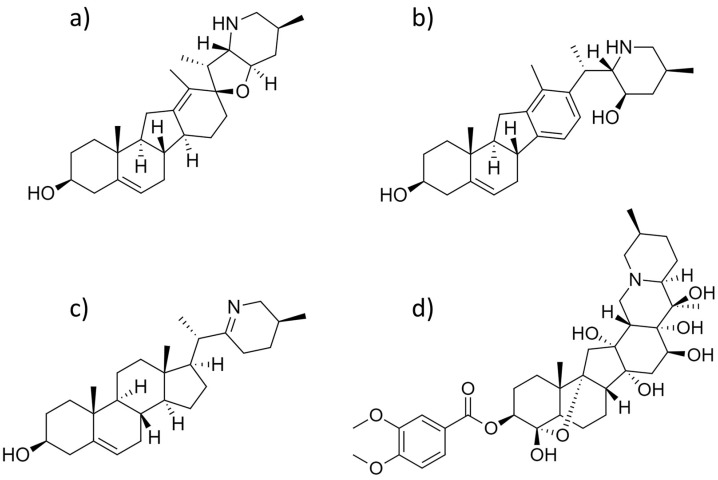
Chemical structures of (**a**) cyclopamine, (**b**) veratramine, (**c**) verazine, and (**d**) veratridine.

**Figure 5:**
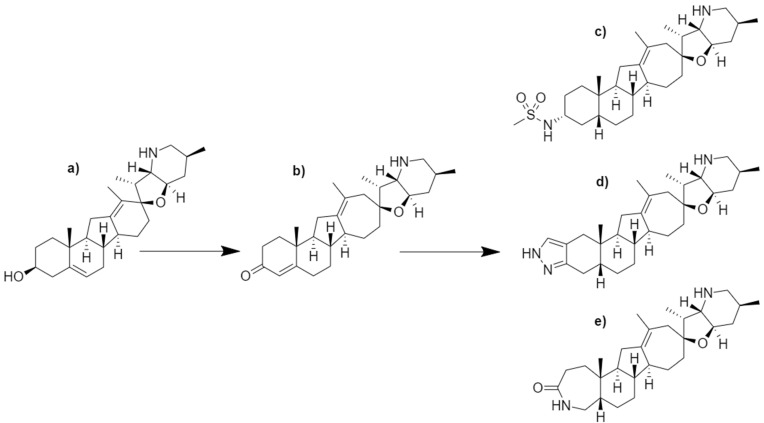
Chemical structures of (**a**) cyclopamine and its semi-synthetic (**b**–**e**) analogs. Initial modifications led to the production of the (**b**) α/β-unsaturated ketone analog with improved chemical stability and aqueous solubility. Successive studies produced three lead compounds: (**c**) methyl sulfonamide analog, (**d**) pyrazole analog, and the (**e**) lactam analog. Compound (**c**) was named saridegib, now known as patidegib.

Cyclopamine (Figure 4a) was isolated from *V. grandiflorum* in 1965 and was the first molecule identified to inhibit the Hedgehog signaling pathway [3,4,6,47]. Since its discovery, cyclopamine has also been identified in *V. californicum* and *V. parviflorum* [6,14,15,42,45]. Cyclopamine is classified as a jervanine-type steroidal alkaloid with a C-nor-D-homosteroidal skeleton, where the C and D rings of the steroidal backbone are five- and six-membered rings, respectively [6]. Jervanine-type alkaloids feature a tetrahydrofuran E-ring that links the nitrogen containing F-ring to the D-ring through a spiro-carbon at the cyclic ether [6]. This compound has been observed to inhibit Hedgehog signaling in Shh Light II cells, inhibit the growth of breast cancer, induce apoptosis in human prostate cancer, increase the expression of death receptor 5 in tumor necrosis factor-related apoptosis-inducing ligand (TRAIL) resistant gastric cancer cells, induce apoptosis and COX-2 overexpression via PKC activation in HEL and TF1a human erythroleukemia cell lines, and induce growth inhibition in human carcinogenesis of cholangiocarcinoma cell lines [42,48,49,50,51,52]. Cyclopamine shows promise as a human chemotherapy; however, it has limited use due to its low solubility in aqueous solutions (~5 µg/mL), and instability in acidic conditions [53,54]. To addresses these limitations, semi-synthetic approaches have been undertaken to increase potency and solubility [54,55]. The cyclopamine derivative KAAD-cyclopamine included the addition of a 3-keto,*N*-aminoethyl aminocaproyl dihydrocinnamoyl (KAAD) functional group to the F-ring nitrogen; this resulted in a 10-20 fold increase in potency [53,55]. A cyclopamine-tartrate salt was developed to increase the solubility of the compound in water [53,56]. The cyclopamine-tartrate salt was more soluble in water at about 5 mg/mL, had a higher LD_50_ of 62.5 mg/kg of body weight compared to that of cyclopamine, which is 43.5 mg/kg, and had a lower tumor area value in Krt6a-cre: Ptch1*^neo/neo^* mice [56]. Cyclopamine has been used as a molecular scaffold for the semi-synthetic derivative patidegib (Figure 5), which is currently undergoing phase III clinical trials. Patidegib, formerly known as saridegib and IPI-926, has received orphan drug approval for the treatment of nevoid basal cell carcinoma [5,57].

Patidegib was developed through a series of structure–activity relationship (SAR) studies focused on the improvement of potency, aqueous solubility, and chemical stability for cyclopamine [54,58]. In acidic environments, cyclopamine readily converts to veratramine due to the acid-catalyzed opening of the spirotetrahydrofuran E-ring and aromatization of the D-ring [54]. Veratramine possesses the ability to cause neurotoxic effects and hemolysis; thus, removing the potential degradation of cyclopamine, or its analogues, was advantageous to furthering the modification of the cyclopamine skeleton [54,59,60,61]. Tremblay et al. (2008) describe two modifications, a D-ring expansion and the formation of an α/β-unsaturated ketone, to cyclopamine [54]. These modifications not only improved chemical stability in simulated gastric fluid from 60% remaining to 98% remaining following a 60 min incubation, but also maintained Hh inhibitory properties equivalent to cyclopamine [54]. This analog (Figure 5b) was administered to CD-1 mice orally and intravenously resulting in an observed 80% oral bioavailability and elimination half-life of 3.2 h. A successive study by Tremblay et al. (2009) sought to improve upon the previous study by modifying the A-ring system [58]. Although the cyclopamine analog developed in 2008 exhibited improved chemical stability and aqueous solubility, the α/β-unsaturated ketone in the A-ring was observed to be readily metabolized [58]. Through exploration of SAR surrounding the A-ring, three lead compounds emerged with improved bioactivity and metabolic stability (Figure 5c–e). A *cis*-ring fusion system showed an improved bioactivity while the addition of the sulfonamide (Figure 5c), pyrazole (Figure 5d), and lactam (Figure 5e) functionalities increased metabolic stability. All three lead compounds outperformed the previous analog (Figure 5b); however, the sulfonamide containing analog, now known as patidegib, outperformed the other compounds regarding efficacy and pharmacokinetics [58].

#### 2.4.2. Veratramine

Veratramine (Figure 4b) has been identified in *V. parviflorum*, *V. viride*, *V. oxysepalum*, *V. nigrum* L., *V. californicum*, and *V. grandiflorum* [15,42,62,63,64,65]. Similar to cyclopamine, veratramine contains a C-nor-D-homosteroidal skeleton; however, it is further categorized as a veratramine-type alkaloid [6]. In comparison to jervanine-type alkaloids, those of the veratranine-type feature an aromatized D-ring and lack a tetrahydrofuran E-ring that connects the piperidine ring to the D-ring. Veratramine is a lipid-soluble alkaloid that exhibits a range of bioactivities [66]. This alkaloid was observed to cause DNA damage in the cerebellum and cerebral cortex of mice in a dose-dependent trend through the generation of reactive oxygen species; moreover, it inhibited Hedgehog signaling in Shh light II cells, reduced the growth, proliferation, and migration of the PC-3 human metastatic prostate cancer cell line, and induced autophagy-mediated apoptosis by inhibiting PI3K/Akt/mTOR signaling in HepG2 cells [14,42,66,67,68,69]. One study regarding the metabolism of veratramine in male Sprague–Dawley rats suggested that elimination of the alkaloid primarily occurred through phenyl mono-oxidation, hydroxylation, and methylation [66]. The phenyl-oxidation metabolite of veratramine was proposed to lead to the formation of reactive oxygen species that oxidize DNA and proteins [66].

#### 2.4.3. Verazine

Verazine (Figure 4c) is a precursor to steroidal alkaloids, including cyclopamine and veratramine, found across the *Melanthiaceae* and *Solanaceae* plant families [6,15,70,71,72,73,74]. This compound is classified as a verazine-type steroidal alkaloid in the cyclopentanophenanthrene skeleton ring system [6]. The cyclopentanophenanthrene skeleton features a ring scaffold typical of cholesterol, where the C-ring and D-ring are six- and five-membered, respectively [6]. Verazine-type alkaloids are differentiated from additional cyclopentanophenanthrene skeleton alkaloids by the presence of an imine-containing ring [6]. The importance of verazine to the biosynthesis of *Veratrum* steroidal alkaloids has promoted efforts to elucidate its biosynthetic production from cholesterol [74]. Augustin et al. identified cholesterol 22-hydroxylase (CYP90B27), 22-hydroxycholesterol, 26-hydroxylase/oxidase (CYP94N1), 22-hydroxycholesterol-26-al transaminase (GABAT1), and 22-hydroxy-26-aminocholesterol 22-oxidase (CYP90G1) as the four enzymes that transform cholesterol into verazine [74]. Although these efforts illustrated how verazine forms, proceeding steps in the biosynthetic formation of additional steroidal alkaloids remain largely unexplored. Kaneko et al. performed a series of studies that observed the conversion of products within the biosynthetic pathway; however, the mechanisms in which these conversions take place remain unknown [75,76,77,78,79,80,81,82]. Verazine has not been studied for potential anticancer properties, however, the alkaloid does exhibit antifungal and melanogenesis inhibitory properties [72,73]. The growth of *Candida albicans* and *Trichophyton rubrum* was inhibited at minimum inhibitory concentrations of 6.2 µg/mL and 3.1 µg/mL, respectively [74]. Furthermore, melanogenesis in B16 F1 mouse melanoma cells were inhibited with an IC_50_ of <1 µg/mL [72]. Verazine also showed inhibitory activity for Sc7 yeast; however, it proved to be cytotoxic in an M-109 cell line with an IC_50_ of 12.5 µg/mL [70].

#### 2.4.4. Veratridine

Veratridine (Figure 4d) has been identified in *V. album*, *V. viride*, *V. parviflorum*, and *Schoenocaulon officinale* [15,31,36,83]. This compound is classified as a cevanine-type alkaloid with a C-nor-D-homosteroidal skeleton [6]. The cevanine alkaloids are defined by the presence of a six-membered E-ring, being highly hydroxylated, and a hemiketal linkage between C4 and C9 [6]. This compound is primarily recognized as one of the major alkaloids contributing to the cardiotoxic effects from *Veratrum* poisoning [15,27,31,84]. Veratridine has been identified as an agonist of voltage-gated sodium ion channels [85]. The compound binds to the type 2 receptor of voltage-gated sodium ion channels, leading to membrane depolarization and repetitive firing of the nerve [33,35,36,85]. Unlike cyclopamine and veratramine, veratridine has not been observed to inhibit Hedgehog signaling through the antagonism of Smo. Belgacem and Borodinsky used veratridine to study the effects of a Ca^2+^ spike on Gli transcriptional activity [86]. Veratridine selectively inhibits voltage-gated Na^+^ ion channels, resulting in an increase of Ca^2+^ spike activity and diminished Gli levels; in turn, this downregulates Sonic hedgehog (Shh) signaling [86]. In contrast, if voltage-gated Na^+^ and Ca^2+^ ion channels were blocked, Gli transcriptional activity would increase, resulting in the upregulation of Shh signaling [86]. These results suggested that Shh signaling may be selectively regulated by a *Veratrum* steroidal alkaloid in mechanisms other than Smo antagonism.

## 3. Conclusions

*Veratrum* spp. have been investigated for their potential to inhibit the growth of cancers, such as basal cell carcinoma and acute myeloid leukemia, resulting from aberrant activation of the Hedgehog signaling pathway. Although species including *V. viride* provide precedent for the phytochemistry of *V. parviflorum*, only four alkaloids (cyclopamine, veratramine, verazine, and veratridine) with known bioactivities have been identified in *V. parviflorum*. However, over forty-three prominent peaks can be observed in the chromatogram of its ethanolic extract [15]. A combined effort to characterize these unknown alkaloids and assess their capability to antagonize Hh signaling may yield a compound suitable for further study.

Although these compounds have undergone extensive study over the last century, there are gaps in current research that limit the development of *Veratrum* steroidal alkaloids. Few alkaloids may be found in online databases with up-to-date spectral data. With each *Veratrum* spp. containing >100 alkaloids and approximately 20% of those alkaloids receiving full characterization, reducing the likelihood of repeated discovery would make the natural product drug discovery process more efficient. Lu et al. utilized ITS2 sequence and metabolite profiling of *Veratrum* steroidal alkaloids to distinguish species [87]. A continuation of such efforts may prove fruitful for the rapid identification of alkaloids within *Veratrum* biomass extracts. An additional deficiency in current *Veratrum* research is with respect to the biosynthesis of the structurally complex alkaloids. There may be a demand in the future for low-cost production of cyclopamine if the semi-synthetic drug patidegib receives full approval by the FDA. The chemical synthesis of cyclopamine is relatively complex and low yield. Giannis et al. reported a diastereoselective and biomimetic synthetic procedure for cyclopamine with over twenty steps and a 1% overall yield [88]. Furthermore, efforts to cultivate *Veratrum* proved challenging due to the slow growth rate, temperature requirements, and low germination rates [6]. Only four enzymes that catalyze the biosynthesis of cyclopamine have been characterized, leaving the majority of the pathway unknown [74]. Next-generation sequencing technology has been applied to *V. nigrum* to identify candidate genes involved in the biosynthesis of the *Veratrum* steroidal alkaloid jervine [89]. Such technologies may be beneficial for works dedicated to cyclopamine biosynthesis.

## Figures and Tables

**Table 1:** A comparison of the identified steroidal alkaloids and traditional medicinal applications for several *Veratrum* species.

*Veratrum* spp.	Alkaloids Identified	Traditional Medical Applications	References
*V. nigrum* ^1^	Epiverazine, veratramine, and verazine.	Apoplexy, wind-type dysentery, jaundice, scabies, and chronic malaria.	[10,14]
*V. album* ^2^	Verazine, jervine, pseudojervine, rubijervine, veralosine, veralosidine, verabenzoamine, veratroilzigadenine, 15-O-(2-methylbutyroyl)germine, veralosinine, veratramine, veratridine, and cevadine.	Head lice, scabies, neuralgic pain, eczema, fever, hypodermatosis, rheumatism, and headache.	[11,12,13,14]
*V. viride* ^2^	Veratramine, isorubijervosine, pseudojervine, and rubijervine.	Rheumatism, venereal diseases, and analgesic.	[6,12,14]
*V. californicum* ^2^	Cyclopamine, veratramine, muldamine, isorubijervine, cycloposine, and veratrosine.	None reported.	[6,14]
*V. parvilforum* ^1^	Cyclopamine, veratramine, veratridine, and verazine.	None reported.	[15]

^1^ Species within *Veratrum* sect. *Fuscoveratrum* [Clade C]. ^2^ Species within *Veratrum* sect. *Veratrum* [Clade B].

**Table 2:** A summary of several cases of *Veratrum* poisoning, including the causative plant, symptoms, and treatment.

*Veratrum* spp. Ingested	Symptoms	Treatment	References
*V. parviflorum*	Nausea, vomiting, hypotension, and bradycardia.	Antiemetics, intravenous fluid resuscitation, and digoxin immune Fab.	[15]
*V. viride*	Nausea, vomiting, diaphoresis, lightheadedness, bilateral retrobulbar headache, leg spasms, hypotension, bradycardia, paresthesia, dyspnea, and sluggishly reactive 2–3 mm pupils.	Intravenous fluid resuscitation, atropine, promethazine, and dopamine infusion.	[23,24,26,32]
*V. album*	Nausea, vomiting, headache, diarrhea, bradycardia, dizziness, paresthesia, blurred vision, abdominal pain, clouded consciousness, pyrosis, atrioventricular dissociation, and death.	Activated charcoal, antiemetics, intravenous fluid resuscitation, thiethylperazine, atropine, prednisolone, hydrocortisone, tocopherol, unithiol, and digoxin immune Fab.	[25,27,28,29,30,31]

## References

[1] Kuruppu A.I., Paranagama P., Goonasekara C.L. (2019). Medicinal plants commonly used against cancer in traditional medicine formulae in Sri Lanka. Saudi Pharm. J..

[2] Seca A.M.L., Pinto D.C.G.A. (2018). Plant Secondary Metabolites as Anticancer Agents: Successes in Clinical Trials and Therapeutic Application. Int. J. Mol. Sci..

[3] Chen J.K., Taipale J., Cooper M.K., Beachy P.A. (2002). Inhibition of Hedgehog signaling by direct binding of cyclopamine to Smoothened. Genes Dev..

[4] Masamune T., Mori Y., Takasugi M., Murai A., Ohuchi S., Sato N., Katsui N. (1965). 11-Deoxojervine, a new alkaloid from *Veratrum* species. Bull. Chem. Soc. Jpn..

[5] Jamieson C., Martinelli G., Papayannidis C., Cortes J.E. (2020). Hedgehog Pathway Inhibitors: A New Therapeutic Class for the Treatment of Acute Myeloid Leukemia. Blood Cancer Discov..

[6] Chandler C.M., McDougal O.M. (2014). Medicinal history of North American *Veratrum*. Phytochem. Rev..

[7] Zomlefer W.B., Whitten W.M., Williams N.H., Judd W.S. (2003). An overview of *Veratrum* sl (Liliales: Melanthiaceae) and an infrageneric phylogeny based on ITS sequence data. Syst. Bot..

[8] Liao W.-J., Yuan Y.-M., Zhang D.-Y. (2007). Biogeography and evolution of flower color in *Veratrum* (Melanthiaceae) through inference of a phylogeny based on multiple DNA markers. Plant Syst. Evol..

[9] Li H.-J., Jiang Y., Li P. (2006). Chemistry, bioactivity and geographical diversity of steroidal alkaloids from the Liliaceae family. Nat. Prod. Rep..

[10] Li H.l., Tang J., Liu R., Lin M., Wang B., Lv Y., Huang H., Zhang C., Zhang W. (2007). Characterization and identification of steroidal alkaloids in the Chinese herb *Veratrum nigrum* L. by high-performance liquid chromatography/electrospray ionization with multi-stage mass spectrometry. Rapid Commun. Mass Spectrom..

[11] Shikov A.N., Narkevich I.A., Flisyuk E.V., Luzhanin V.G., Pozharitskaya O.N. (2021). Medicinal plants from the 14th edition of the Russian Pharmacopoeia, recent updates. J. Ethnopharmacol..

[12] Alm T. (2002). Norwegian and Sámi Ethnobotany of *Veratrum album* (Melanthiaceae). SIDA Contrib. Bot..

[13] Rätsch C., Hofmann A. (2005). The Encyclopedia of Psychoactive Plants: Ethnopharmacology and Its Applications.

[14] Dirks M.L., Seale J.T., Collins J.M., McDougal O.M. (2021). Review: *Veratrum californicum* Alkaloids. Molecules.

[15] Anwar M., Turner M., Farrell N., Zomlefer W.B., McDougal O.M., Morgan B.W. (2018). Hikers poisoned: *Veratrum* steroidal alkaloid toxicity following ingestion of foraged *Veratrum parviflorum*. Clin. Toxicol..

[16] Zomlefer W.B. (1997). The genera of Melanthiaceae in the southeastern United States. Harv. Pap. Bot..

[17] Watson S. (1878). Contributions to American Botany: Revision of the North American Liliaceae; Descriptions of Some New Species of North American Plants. Proc. Am. Acad. Arts Sci..

[18] Thompson Y., D’Angelo E., Karathanasis A., Sandefur B.C. (2012). Plant community composition as a function of geochemistry and hydrology in three Appalachian wetlands. Ecohydrology.

[19] Brundage S., Lady Bird Johnson Wildflower Center Veratrum parviflorum. https://www.wildflower.org/gallery/result.php?id_image=66638.

[20] Annable C. (2022). Melanthium Parviflorum.

[21] Baskin C.C., Baskin J.M. (1988). Germination Ecophysiology of Herbaceous Plant Species in a Temperate Region. Am. J. Bot..

[22] Atwood E.L. (1941). White-tailed deer foods of the United States. J. Wildl. Manag..

[23] Jaffe A.M., Gephardt D., Courtemanche L. (1990). Poisoning due to ingestion of *Veratrum viride* (false hellebore). J. Emerg. Med..

[24] Prince L., Stork C. (2000). Prolonged cardiotoxicity from poison lilly (*Veratrum viride*). Vet. Hum. Toxicol..

[25] Zagler B., Zelger A., Salvatore C., Pechlaner C., Giorgi F.D., Wiedermann C.J. (2005). Dietary poisoning with *Veratrum album*—A report of two cases. Wien. Klin. Wochenschr..

[26] Forrester J.D., Price J.H., Holstege C.P. (2010). Intoxication with a Ramp (*Allium tricocca*) Mimicker. Wilderness Environ. Med..

[27] Gilotta I., Brvar M. (2010). Accidental Poisoning with Veratrum album Mistaken for Wild Garlic (*Allium ursinum**)*. Clin. Toxicol..

[28] Melnik E.V., Belova M.V., Potskhveriya M.M., Simonova A.Y., Tyurin I.A., Ramenskaya G.V. (2022). *Veratrum* Alkaloid Determination in Four Cases of *Veratrum* Aqua Poisonings. J. Anal. Toxicol..

[29] Festa M., Andreetto B., Ballaris M.A., Panio A., Piervittori R. (1996). A Case of Veratrum Poisoning. Minerva Anestesiol..

[30] Grobosch T., Binscheck T., Martens F., Lampe D. (2008). Accidental Intoxication with *Veratrum album*. J. Anal. Toxicol..

[31] Gaillard Y., Pepin G. (2001). LC-EI-MS Determination of Veratridine and Cevadine in Two Fatal Cases of *Veratrum* album Poisoning. J. Anal. Toxicol..

[32] Bechtel L., Lawrence D., Haverstick D., Powers J., Wyatt S., Croley T., Holstege C. (2010). Ingestion of False Hellebore Plants Can Cross-React with a Digoxin Clinical Chemistry Assay. Clin. Toxicol..

[33] Schep L.J., Schmierer D.M., Fountain J.S. (2006). *Veratrum* Poisoning. Toxicol. Rev..

[34] Turner M.W., Rossi M., Campfield V., French J., Hunt E., Wade E., McDougal O.M. (2019). Steroidal alkaloid variation in *Veratrum californicum* as determined by modern methods of analytical analysis. Fitoterapia.

[35] Wang S.Y., Wang G.K. (2003). Voltage-gated sodium channels as primary targets of diverse lipid-soluble neurotoxins. Cell. Signal..

[36] Ulbricht W. (1998). Effects of Veratridine on Sodium Currents and Fluxes. Reviews of Physiology, Biochemistry and Pharmacology.

[37] Jarisch A., Richter H. (1939). Die Kreislaufwirkung des Veratrins. Archiv f. experiment. Pathol. Pharmakol..

[38] Schaefer H. (1950). Elektrophysiologie der Herznerven. Rev. Physiol. Biochem. Pharmacol..

[39] von Bezold A. (1867). Uber die Physiologischen Wirkungen des Essigsauren Veratrins. Unters. Aus. Dem. Physiol. Lab. Wurzbg..

[40] Cooper M.K., Porter J.A., Young K.E., Beachy P.A. (1998). Teratogen-Mediated Inhibition of Target Tissue Response to Shh Signaling. Science.

[41] Gao L., Chen F., Li X., Xu S., Huang W., Ye Y. (2016). Three New Alkaloids from *Veratrum grandiflorum* Loes with Inhibition Activities on Hedgehog Pathway. Bioorganic Med. Chem. Lett..

[42] Turner M.W., Cruz R., Elwell J., French J., Mattos J., McDougal O.M. (2018). Native V. californicum Alkaloid Combinations Induce Differential Inhibition of Sonic Hedgehog Signaling. Molecules.

[43] Binns W., James L.F., Shupe J.L., Thacker E.J. (1962). Cyclopian-Type Malformation in Lambs. Arch. Environ. Health.

[44] Binns W., James L.F., Shupe J.L. (1964). Toxicosis of *Veratrum californicum* in Ewes and its Relationship to a Congenital Deformity in Lambs. Ann. N. Y. Acad. Sci..

[45] Keeler R.F., Binns W. (1968). Teratogenic Compounds of *Veratrum californicum* (Durand) V. Comparison of Cyclopian Effects of Steroidal Alkaloids from the Plant and Structurally Related Compounds from Other Sources. Teratology.

[46] Deshpande I., Liang J., Hedeen D., Roberts K.J., Zhang Y., Ha B., Latorraca N.R., Faust B., Dror R.O., Beachy P.A. (2019). Smoothened Stimulation by Membrane Sterols Drives Hedgehog Pathway Activity. Nature.

[47] Incardona J.P., Gaffield W., Kapur R.P., Roelink H. (1998). The teratogenic *Veratrum* alkaloid cyclopamine inhibits sonic hedgehog signal transduction. Development.

[48] El Khatib M., Kalnytska A., Palagani V., Kossatz U., Manns M.P., Malek N.P., Wilkens L., Plentz R.R. (2013). Inhibition of Hedgehog Signaling Attenuates Carcinogenesis In Vitro and Increases Necrosis of Cholangiocellular Carcinoma. Hepatology.

[49] Na Y.J., Lee D.H., Kim J.L., Kim B.R., Park S.H., Jo M.J., Jeong S., Kim H.J., Lee S.Y., Jeong Y.A. (2017). Cyclopamine Sensitizes TRAIL-Resistant Gastric Cancer Cells to TRAIL-Induced Apoptosis Via Endoplasmic Reticulum Stress-Mediated Increase of Death Receptor 5 and Survivin Degradation. Int. J. Biochem. Cell. Biol..

[50] Shaw G., Price A.M., Ktori E., Bisson I., Purkis P.E., McFaul S., Oliver R.T., Prowse D.M. (2008). Hedgehog Signalling in Androgen Independent Prostate Cancer. Eur. Urol..

[51] Zhang X., Harrington N., Moraes R.C., Wu M.-F., Hilsenbeck S.G., Lewis M.T. (2009). Cyclopamine Inhibition of Human Breast Cancer Cell Growth Independent of Smoothened (Smo). Breast Cancer Res. Treat..

[52] Ghezali L., Leger D.Y., Limami Y., Cook-Moreau J., Beneytout J.L., Liagre B. (2013). Cyclopamine and Jervine Induce COX-2 Overexpression in Human Erythroleukemia Cells but Only Cyclopamine has a Pro-Apoptotic Effect. Exp. Cell. Res..

[53] Lee S.T., Welch K.D., Panter K.E., Gardner D.R., Garrossian M., Chang C.-W.T. (2014). Cyclopamine: From Cyclops Lambs to Cancer Treatment. J. Agric. Food Chem..

[54] Tremblay M.R., Nevalainen M., Nair S.J., Porter J.R., Castro A.C., Behnke M.L., Yu L.-C., Hagel M., White K., Faia K. (2008). Semisynthetic Cyclopamine Analogues as Potent and Orally Bioavailable Hedgehog Pathway Antagonists. J. Med. Chem..

[55] Taipale J., Chen J.K., Cooper M.K., Wang B., Mann R.K., Milenkovic L., Scott M.P., Beachy P.A. (2000). Effects of Oncogenic Mutations in Smoothened and Patched can be Reversed by Cyclopamine. Nature.

[56] Fan Q., Gu D., He M., Liu H., Sheng T., Xie G., Li C.-X., Zhang X., Wainwright B., Garrossian A. (2011). Tumor 61 Shrinkage by Cyclopamine Tartrate through Inhibiting Hedgehog Signaling. Chin. J. Cancer.

[57] US Food and Drug Administration Orphan Drug Designations and Approvals. https://www.accessdata.fda.gov/scripts/opdlisting/oopd/detailedIndex.cfm?cfgridkey=587917.

[58] Tremblay M.R., Lescarbeau A., Grogan M.J., Tan E., Lin G., Austad B.C., Yu L.-C., Behnke M.L., Nair S.J., Hagel M. (2009). Discovery of a Potent and Orally Active Hedgehog Pathway Antagonist (IPI-926). J. Med. Chem..

[59] Badria F.A., McChesney J.D., Halim A.F., Zaghloul A.M., El Sayed K.A. (1995). Time course and inhibition of stavaroside K, veratramine and cevine-induced hemolysis by other pregnane glycosides and *Veratrum* alkaloids. Pharmazie.

[60] Nagata R., Izumi K. (1991). Veratramine-induced behavior associated with serotonergic hyperfunction in mice. Jpn. J. Pharmacol..

[61] Thron C.D., McCann F.V. (1999). Pharmacological tests of the mechanism of the periodic rhythm caused by veratramine in the sinoatrial node of the guinea pig. Gen. Pharmacol..

[62] El Sayed K.A., McChesney J.D., Halim A.F., Zaghloul A.M., Lee I.-S. (1996). A Study of Alkaloids in *Veratrum* viride Aiton. Int. J. Pharmacogn..

[63] Saito K. (1940). Veratramine, a New Alkaloid of White Hellebore (*Veratrum grandifiorum* Loes. fil.). Bull. Chem. Soc. Jpn..

[64] Wang L., Li W., Liu Y. (2008). Hypotensive Effect and Toxicology of Total Alkaloids and Veratramine from Roots and Rhizomes of *Veratrum nigrum* L. in Spontaneously Hypertensive Rats. Pharmazie.

[65] Yu Y., Li H., Jiang Y. (2014). Separation and Preparation of Five Cyclopamine Analogs from Rhizomes of *Veratrum oxysepalum* Turcz. by Two-Step HighSpeed Counter-Current Chromatography. Sep. Sci. Technol..

[66] Cong Y., Guo J., Tang Z., Lin S., Zhang Q., Li J., Cai Z. (2014). Metabolism Study of Veratramine Associated with Neurotoxicity by Using HPLC–MSn. J. Chromatogr. Sci..

[67] Turner M.W., Cruz R., Mattos J., Baughman N., Elwell J., Fothergill J., Nielsen A., Brookhouse J., Bartlett A., Malek P. (2016). Cyclopamine Bioactivity by Extraction Method from *Veratrum californicum*. Bioorg. Med. Chem..

[68] Khanfar M.A., El Sayed K.A. (2013). The *Veratrum* Alkaloids Jervine, Veratramine, and their Analogues as Prostate Cancer Migration and Proliferation Inhibitors: Biological Evaluation and Pharmacophore Modeling. Med. Chem. Res..

[69] Yin L., Xia Y., Xu P., Zheng W., Gao Y., Xie F., Ji Z. (2020). Veratramine suppresses human HepG2 liver cancer cell growth in vitro and in vivo by inducing autophagic cell death. Oncol. Rep..

[70] Abdel-Kader M.S., Bahler B.D., Malone S., Werkhoven M.C., van Troon F., David I., Wisse J.H., Bursuker I., Neddermann K.M., Mamber S.W. (1998). DNA-damaging steroidal alkaloids from Eclipta alba from the suriname rainforest. J. Nat. Prod..

[71] Colmenares A.P., Alarcón L., Rojas L.B., Mitaine-Offer A.-C., Pouységu L., Quideau S., Paululat T., Usubillaga A., Lacaille-Dubois M.-A. (2010). New steroidal alkaloids from Solanum hypomalacophyllum. Nat. Prod. Commun..

[72] Kim H.-J., Kang S.-J., Kang S.-H., Kim C.-H., Jung M.-H., Jin M.-H. (2002). Three melanogenesis inhibitors from the roots of *Veratrum nigrum*. Korean J. Pharmacogn..

[73] Kusano G., Takahashi A., Sugiyama K., Nozoe S. (1987). Antifungal properties of Solanum alkaloids. Chem. Pharm. Bull..

[74] Augustin M.M., Ruzicka D.R., Shukla A.K., Augustin J.M., Starks C.M., O’Neil-Johnson M., McKain M.R., Evans B.S., Barrett M.D., Smithson A. (2015). Elucidating steroid alkaloid biosynthesis in *Veratrum californicum*: Production of verazine in Sf9 cells. Plant J..

[75] Kaneko K., Kawamura N., Kuribayashi T., Tanaka M., Mitsuhashi H., Koyama H. (1978). Structures of two cevanine alkaloids, shinonomenine and veraflorizine, and a cevanidane alkaloid, procevine, isolated from illuminated *Veratrum*. Tetrahedron Lett..

[76] Kaneko K., Kawamura N., Mitsuhashi H., Ohsaki K. (1979). Two new *Veratrum* alkaloids, hosukinidine and epirubijervine from illuminated *Veratrum* plant. Chem. Pharm. Bull..

[77] Kaneko K., Mitsuhashi H., Hirayama K., Ohmori S. (1970). 11-Deoxojervine as a precursor for jervine biosynthesis in *Veratrum grandiflorum*. Phytochemistry.

[78] Kaneko K., Mitsuhashi H., Hirayama K., Yoshida N. (1970). Biosynthesis of C-nor-D-homo-steroidal alkaloids from acetate-1-14C, cholesterol-4-14C and cholesterol-26-14C in *Veratrum grandiflorum*. Phytochemistry.

[79] Kaneko K., Seto H., Motoki C., Mitsuhashi H. (1975). Biosynthesis of rubijervine in *Veratrum grandiflorum*. Phytochemistry.

[80] Kaneko K., Tanaka M.W., Mitsuhashi H. (1976). Origin of nitrogen in the biosynthesis of solanidine by *Veratrum grandiflorum*. Phytochemistry.

[81] Kaneko K., Tanaka M.W., Mitsuhashi H. (1977). Dormantinol, a possible precursor in solanidine biosynthesis, from budding *Veratrum grandiflorum*. Phytochemistry.

[82] Kaneko K., Watanabe M., Taira S., Mitsuhashi H. (1972). Conversion of solanidine to jerveratrum alkaloids in *Veratrum grandiflorum*. Phytochemistry.

[83] Maison G.L., Gotz E., Stutzman J.W. (1951). Relative Hypotensive Activity of Certain *Veratrum* Alkaloids. J. Pharmacol. Exp. Ther..

[84] Taniguchi M., Minatani T., Miyazaki H., Tsuchihashi H., Zaitsu K. (2021). A highly sensitive quantification method for 12 plant toxins in human serum using liquid chromatography tandem mass spectrometry with a quick solid-phase extraction technique. J. Pharm. Biomed. Anal..

[85] de Lera Ruiz M., Kraus R.L. (2015). Voltage-Gated Sodium Channels: Structure, Function, Pharmacology, and Clinical Indications. J. Med. Chem..

[86] Belgacem Y.H., Borodinsky L.N. (2015). Inversion of Sonic hedgehog action on its canonical pathway by electrical activity. Proc. Natl. Acad. Sci. USA.

[87] Lu Q., Wang S., Yin Z., Chen Q., He X., Wang Q., Hu Q., Gu Y., Tang H., Xie H. (2022). Identification of *Veratrum* Species in Pimacao Based on ITS2 Sequences and Steroidal Alkaloids by a Pseudo-Targeted Metabolomics Method. Front. Plant Sci..

[88] Giannis A., Heretsch P., Sarli V., Stößel A. (2009). Synthesis of Cyclopamine Using a Biomimetic and Diastereoselective Approach. Angew. Chem. Int. Ed. Engl..

[89] Szeliga M., Ciura J., Tyrka M. (2020). Representational Difference Analysis of Transcripts Involved in Jervine Biosynthesis. Life.

